# Highly Pathogenic Avian Influenza A(H7N3) Virus in Poultry Workers, Mexico, 2012

**DOI:** 10.3201/eid1909.130087

**Published:** 2013-09

**Authors:** Irma Lopez-Martinez, Amanda Balish, Gisela Barrera-Badillo, Joyce Jones, Tatiana E. Nuñez-García, Yunho Jang, Rodrigo Aparicio-Antonio, Eduardo Azziz-Baumgartner, Jessica A. Belser, José E. Ramirez-Gonzalez, Janice C. Pedersen, Joanna Ortiz-Alcantara, Elizabeth Gonzalez-Duran, Bo Shu, Shannon L. Emery, Mee K. Poh, Gustavo Reyes-Teran, Joel A. Vazquez-Perez, Santiago Avila-Rios, Timothy Uyeki, Stephen Lindstrom, Julie Villanueva, Jerome Tokars, Cuitláhuac Ruiz-Matus, Jesus F. Gonzalez-Roldan, Beverly Schmitt, Alexander Klimov, Nancy Cox, Pablo Kuri-Morales, C. Todd Davis, José Alberto Diaz-Quiñonez

**Affiliations:** Instituto de Diagnóstico y Referencia Epidemiológicos, Mexico City, Mexico (I. Lopez-Martinez, G. Barrera-Badillo, T.E. Nuñez-García, R. Aparicio-Antonio, J.E. Ramirez-Gonzalez, J. Ortiz-Alcantara, E. Gonzalez-Duran, J.A. Diaz-Quiñonez);; Centers for Disease Control and Prevention, Atlanta, Georgia, USA (A. Balish, J. Jones, Y. Jang, E. Azziz-Baumgartner, J.A. Belser, B. Shu, S.L. Emery, M.K. Poh, T. Uyeki, S. Lindstrom, J. Villanueva, J. Tokars, A. Klimov, N. Cox, C.T. Davis);; US Department of Agriculture, Ames, Iowa, USA (J.C. Pedersen, B. Schmitt);; Instituto Nacional de Enfermedades Respiratorias, Mexico City (G. Reyes-Teran, J.A. Vazquez-Perez, S. Avila-Rios);; Dirección General de Epidemiología, Mexico City (C. Ruiz-Matus, J.F. Gonzalez-Roldan);; Subsecretaría de Prevención y Promoción de la Salud, Mexico City (P. Kuri-Morales)

**Keywords:** influenza virus, H7N3, highly pathogenic avian influenza A virus, viruses, conjunctivitis, poultry workers, Mexico

## Abstract

We identified 2 poultry workers with conjunctivitis caused by highly pathogenic avian influenza A(H7N3) viruses in Jalisco, Mexico. Genomic and antigenic analyses of 1 isolate indicated relatedness to poultry and wild bird subtype H7N3 viruses from North America. This isolate had a multibasic cleavage site that might have been derived from recombination with host rRNA.

Although wild birds might be infected with influenza A(H7) viruses, outbreaks among poultry are rare. Human infection with influenza A(H7) virus is rare but has been documented after direct contact with infected birds (*1*). Conjunctivitis or upper respiratory tract symptoms developed in patients infected with this virus, and outcomes ranged from mild disease to death (*1*,*2*). In North America, 6 persons infected with influenza A(H7) virus have been reported; all patients recovered (*2*–*6*). We report the cases of 2 poultry workers with conjunctivitis caused by highly pathogenic avian influenza (HPAI) A(H7N3) viruses during poultry-related outbreaks in Jalisco, Mexico (*5*).

## The Study

In June 2012, outbreaks of (HPAI) A(H7N3) virus in poultry on farms throughout Jalisco State were reported by the National Service for Health, Safety, and Food Quality in Mexico (*7*,*8*). A 32-year-old poultry worker who reported irritation in her left eye was examined at a clinic in Jalisco on July 7. Bilateral conjunctival swab specimens were collected and sent to the Institute for Epidemiologic Diagnosis and Reference (InDRE) in Mexico City, where H7 subtype virus infection was confirmed by real-time reverse transcription PCR (RT-PCR). HPAI A(H7N3) virus had been suspected because the patient collected eggs on a farm that had had HPAI A (H7N3) virus infection among poultry. The Mexican International Health Regulation authority reported the case to the World Health Organization on July 19.

Several days later, a 52-year-old man, who was related to the first patient and worked on the same farm, visited a local clinic and reported conjunctivitis. Conjunctival swab specimens from this patient were also positive for H7 subtype virus infection by real-time RT-PCR. Both patients were treated symptomatically and recovered without sequelae (*5*). We describe characteristics of the virus isolated from the 32-year-old woman.

Conjunctival swab specimens were placed in virus transport medium and shipped to InDRE for diagnostic testing. RNA from clinical samples was extracted by using the QIAamp Viral RNA Mini Kit (QIAGEN, Valencia, CA, USA) according to the manufacturer’s protocol. Samples were subjected to real-time RT-PCR by using an H7 hemagglutinin (HA) gene–specific assay. Viruses were isolated from RT-PCR–positive clinical samples collected from each eye by inoculating embryonated chicken eggs and incubating them for 48 h before harvest of allantoic fluid. Isolates were sent to the Centers for Disease Control and Prevention (Atlanta, GA, USA), where virus was reisolated in embryonated chicken eggs for further characterization.

Nucleotide sequences of 8 influenza A gene segments from a virus isolate were generated by semiconductor next-generation sequencing with Ion PGM (Life Technologies, Carlsbad, CA, USA) and MBTuni12 and MBTuni13 primers as described (*9*) at InDRE/Instituto Nacional de Enfermedades Respiratori and by RT-PCR of overlapping fragments of each gene by using H7N3 subtype and avian influenza virus–specific primers at the Centers for Disease Control and Prevention. Sequences were aligned and phylogenetic trees were constructed from each gene alignment by using a neighbor-joining approach implemented in MEGA5 (www.megasoftware.net/) with 1,000 bootstrap replicates.

**Figure 1:**
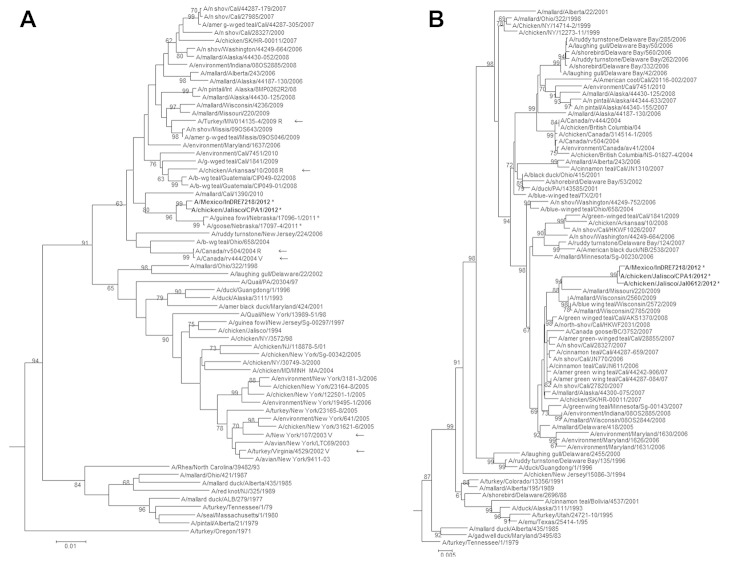
Phylogenetic trees A) of hemagglutinin (HA) and B) neuraminidase (NA) genes of highly pathogenic avian influenza A (H7N3) virus isolated from a poultry worker with conjunctivitis in Jalisco State, Mexico, July 2012, and other influenza viruses. Reassortant vaccine candidates are shown with a V, and hemagglutinin inhibition (HI) reference viruses used in HI tests are shown with an R in the HA tree. Arrows indicate strain names with V and R. Highly pathogenic avian influenza A(H7N3) viruses from Mexico are indicated in **boldface**. Trees were midpoint rooted with other North American-lineage H7 HA and N3 NA gene sequences, and subtrees containing ancestral and related viruses were produced. Bootstrap values >60 are shown above or below branches in each tree. *Viruses detected in 2011 or 2012. Scale bars indicate nucleotide substitutions per site.

**Figure 2:**
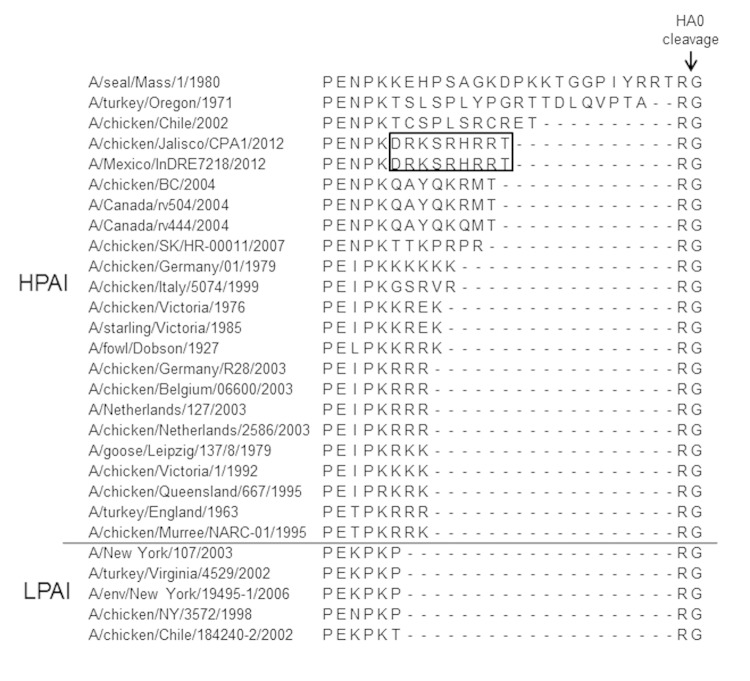
Multibasic cleavage sites of highly pathogenic avian influenza (HPAI) A(H7N3) virus isolated from a poultry worker with conjunctivitis in Jalisco State, Mexico, July 2012, and other influenza viruses. Box indicates novel amino acid cleavage site sequence motif. HA, hemagglutinin; LPAI, low pathogenicity avian influenza. Hyphens indicate gaps in the sequence alignments whereby 1 sequence has an insertion of amino acids relative to shorter sequences.

Genomic sequences confirmed that the conjunctivitis was caused by infection with an HPAI A(H7N3) virus closely related to HPAI A(H7N3) viruses collected during poultry outbreaks in Jalisco State (Figure 1, Appendix). The full genome of 1 isolate was deposited in GenBank under accession no. CY125725–32. Like reported avian A(H7N3) virus sequences from Jalisco, the human isolate had a multibasic cleavage site indicative of an HPAI A virus (*7*) (Figure 2). Genetic similarity of nucleotides at the cleavage site suggested that this region was inserted into the H7 HA gene at the site of HA0 protein cleavage by nonhomologous recombination of host rRNA from an unknown source (*7*). Comparison of this protein sequence motif with other HPAI and low pathogenicity avian influenza (LPAI) H7 viruses showed that this sequence indicated a novel cleavage site not observed in influenza A virus HA gene sequences (Figure 2). However, multiple arginine amino acids in this motif would be predicted to result in a highly pathogenic phenotype in chickens.

Phylogenetic trees of HA and neuraminidase (NA) genes indicated high similarity of HPAI A(H7N3) viruses detected in Mexico and LPAI viruses collected from wild birds and poultry in North America (Figure 1). HA genes clustered with LPAI A(H7N9) viruses from turkeys, geese, and guinea fowl in the United States during 2011 (*10*). The N3 NA genes grouped with LPAI viruses of various subtypes, clustering most closely with viruses collected from wild birds in the midwestern United States in 2009. Internal genes also clustered with LPAI viruses from various subtypes collected primarily in California in 2010 (online Technical Appendix, wwwnc.cdc.gov/EID/article/19/9/13-0087-Techapp1.pdf). An exception was the polymerase acidic gene, which was most closely related to an H11N9 subtype virus from Mississippi.

HA and other protein gene alignments were assessed for putative markers of virulence, mammalian adaptation, receptor-binding specificity, and antiviral drug resistance. Besides the multibasic cleavage site, the virus had typical avian consensus amino acid residues in the HA protein at positions involved in preferential receptor binding to avian sialic acid receptors (amino acids Q226 and G228 by H3 numbering). Avian consensus sequences at other motifs/amino acid positions in proteins of interest were identified, suggesting that the virus had not accumulated described mammalian host adaptive mutations or known virulence markers.

Antigenic characterization was performed by using a panel of ferret antiserum in hemagglutination inhibition (HI) tests with turkey erythrocytes as described (*11*). The HI assay demonstrated relatedness of HPAI A(H7N3) virus with other H7 subtype viruses from North America and a high level of cross-reactivity with the current H7 World Health Organization prepandemic vaccine candidate, A/Canada/rv444/2004, and other North American and Eurasian lineage H7 viruses (Table). Antiserum against HPAI A(H7N3) virus was cross-reactive with North American and Eurasian lineage H7 subtype viruses but showed higher levels of heterologous cross-reactivity with recent H7 viruses collected in the United States and a greater reduction in heterologous titers against Eurasian lineage H7 viruses. Although there were several amino acid differences compared with older North American H7 HA1 protein sequences (27–32 changes), only 5 changes were identified when compared with A/Canada/rv444/2004 virus, indicating a high degree of genetic conservation among this group of H7 viruses.

**Table:** Hemagglutination inhibition titers of North American and Eurasian lineage influenza A (H7) viruses from wild birds, poultry, and humans*

Antigen	Virus and titer								
Reference	CN/444	CN/504	MX/7218	GS/NE	TK/VA	NY/107	TK/MN	NL/219	DK/VN
A/Canada/RV444/2004 H7N3	**80**	320	80	80	80	80	80	160	10
A/Canada/RV504/2004 H7N3	160	**320**	160	160	80	80	160	160	20
A/Mexico/INDRE7218/2012 H7N3	160	320	**160**	160	80	80	160	160	20
A/GS/Nebraska/17097–4/2011 H7N9	160	320	160	**160**	80	160	80	160	20
A/TK/Virginia/4529/2002 H7N2	160	320	160	160	**320**	1,280	20	80	20
A/New York/107/2003 H7N2	160	320	80	160	160	**160**	10	80	20
A/TK/Minnesota/0141354/2009 H7N9	40	80	40	80	20	20	**80**	40	5
A/Netherlands/219/2003 H7N7	20	40	10	40	20	5	40	**160**	10
A/DK/Vietnam/NCVD-197/2009 H7N3	80	160	80	20	5	10	20	40	**80**
Test									
A/Canada/RV444/2004 x PR8 (H7N3)	160	320	80	160	80	80	80	160	20
A/GF/Nebraska/17096–1/2011 (H7N9)	320	640	320	640	160	320	640	320	40
A/CK/Arkansas/10/2008 (H7N3)	160	320	80	160	80	80	320	80	20

*Homologous titers of reference antigen to serum samples are indicated in **boldface**. CN, Canada; MX, Mexico; GS, goose; TK, turkey; NY, New York; NL, The Netherlands; INDRE, Institute for Epidemiologic Diagnosis and Reference; DK, duck; GF, guinea fowl; CK, chicken.

To determine the drug concentration required to inhibit 50% of NA activity, we preformed a functional neuraminidase inhibition (NAI) assay. A fluorescent NAI test was conducted as described (*12*). Oseltamivir-sensitive H1N1 subtype virus (A/Texas/36/91) and its oseltamivir-resistant counterpart with mutation H274Y (N2 numbering) were included as controls. NAI assays showed that the virus was sensitive to neuraminidase inhibitors (zanamivir and oseltamivir). No putative markers of antiviral drug resistance were identified in either NA or matrix genes.

## Conclusions

Emergence of a novel (HPAI) A(H7N3) virus is a reminder of the devastating effect this virus can have on poultry industries and its potential for interspecies transmission. The finding that the HA cleavage site of this virus was probably a result of nonhomologous recombination, as described for other avian influenza A(H7) virus outbreaks, underscores the potential for emergence of HPAI H7 viruses (*13*,*14*). Established mammalian models of ocular infection with H7 subtype influenza A viruses associated with human conjunctivitis demonstrated that these viruses replicated efficiently in eye and respiratory tract tissues (*15*).

Although further studies are needed to investigate in vivo transmissibility of this virus, direct transmission of this virus from infected poultry to humans remains a threat and warrants use of personal protective equipment (including goggles for eye protection) and monitoring persons at risk to prevent additional cases in humans. Health authorities should consider avian influenza A virus infection in patients who have conjunctivitis or influenza-like illness and contact with poultry in areas with known avian influenza outbreaks.

Technical Appendix Phylogenetic trees of polymerase basic 2, polymerase basic 1, polymerase acidic, nucleoprotein, matrix, and nonstructural protein genes of highly pathogenic avian influenza (HPAI) A(H7N3) virus isolated from a poultry worker with conjunctivitis in Jalisco State, Mexico, July 2012, and other influenza viruses. HPAI A(H7N3) viruses from Mexico are indicated in **boldface**. Bootstrap values >60 are shown above or below branches in each tree. *Viruses detected in 2011 or 2012. Scale bars indicate nucleotide substitutions per site.
