# Supplementary material for: Highly Pathogenic Avian Influenza A(H7N3) Virus in Poultry Workers, Mexico, 2012
Source: Emerg Infect Dis. 2013 Sep;19(9):1531–4. doi: 10.3201/eid1909.130087 (PMC3810917; doi:10.3201/eid1909.130087)
Supplement: Technical Appendix — Phylogenetic trees of polymerase basic 2, polymerase basic 1, polymerase acidic, nucleoprotein, matrix, and nonstructural protein genes of highly pathogenic avian influenza (HPAI) A(H7N3) virus isolated from a poultry worker with conjunctivitis in Jalisco State, Mexico, July 2012, and other influenza viruses. HPAI A(H7N3) viruses from Mexico are indicated in boldface. Bootstrap values >60 are shown above or below branches in each tree. *Viruses detected in 2011 or 2012. Scale bars indicate nucleotide substitutions per site. [file 13-0087-Techapp-s1.pdf]

Article DOI: http://dx.doi.org/10.3201/eid1909.130087

Highly Pathogenic Avian Influenza A(H7N3) Virus in Poultry Workers, Mexico, 2012

Technical Appendix

Technical Appendix Figure (following pages). Phylogenetic trees of A) polymerase basic 2, B) polymerase basic 1, C) polymerase acidic, D) nucleoprotein, E) matrix, and F) nonstructural protein genes of highly pathogenic avian influenza (HPAI) A(H7N3) virus isolated from a poultry worker with conjunctivitis in Jalisco State, Mexico, July 2012, and other influenza viruses. HPAI A(H7N3) viruses from Mexico indicated in **boldface**. Bootstrap values >60 are shown above or below branches in each tree. *Indicates viruses detected in 2011 or 2012. Scale bars indicate nucleotide substitutions per site.

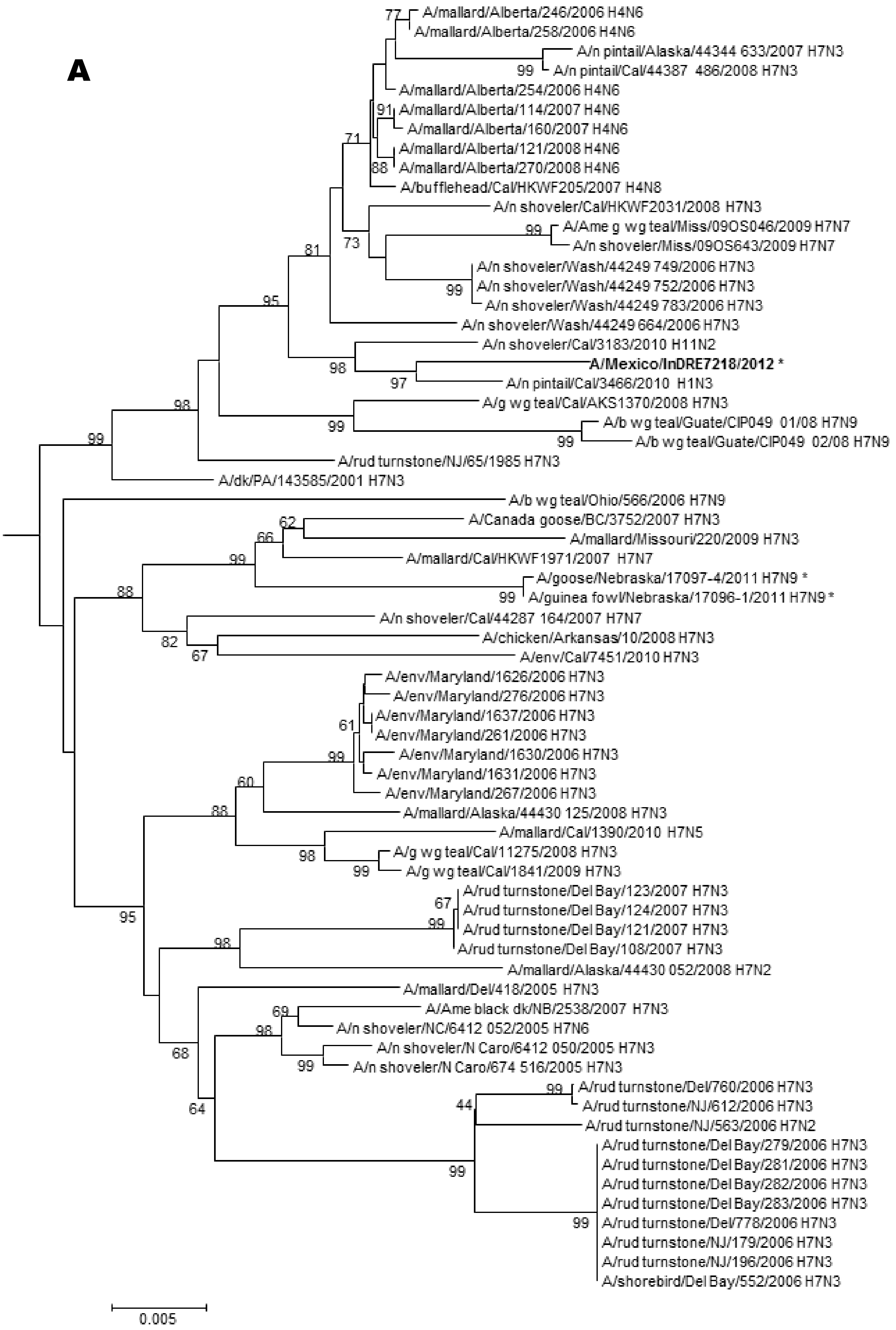
A
A/mallard/Alberta/246/2006 H4N6
A/mallard/Alberta/258/2006 H4N6
A/n pintail/Alaska/44344 633/2007 H7N3
A/n pintail/Cal/44387 486/2008 H7N3
A/mallard/Alberta/254/2006 H4N6
A/mallard/Alberta/114/2007 H4N6
A/mallard/Alberta/160/2007 H4N6
A/mallard/Alberta/121/2008 H4N6
A/mallard/Alberta/270/2008 H4N6
A/bufflehead/Cal/HKWF205/2007 H4N8
A/n shoveler/Cal/HKWF2031/2008 H7N3
A/Ame g wg teal/Miss/09OS046/2009 H7N7
A/n shoveler/Miss/09OS643/2009 H7N7
A/n shoveler/Wash/44249 749/2006 H7N3
A/n shoveler/Wash/44249 752/2006 H7N3
A/n shoveler/Wash/44249 783/2006 H7N3
A/n shoveler/Wash/44249 664/2006 H7N3
A/n shoveler/Cal/3183/2010 H11N2
A/Mexico/InDRE7218/2012 *
A/n pintail/Cal/3466/2010 H1N3
A/g wg teal/Cal/AKS1370/2008 H7N3
A/b wg teal/Guate/CIP049 01/08 H7N9
A/b wg teal/Guate/CIP049 02/08 H7N9
A/rud turnstone/NJ/65/1985 H7N3
A/dk/PA/143585/2001 H7N3
A/b wg teal/Ohio/566/2006 H7N9
A/Canada goose/BC/3752/2007 H7N3
A/mallard/Missouri/220/2009 H7N3
A/mallard/Cal/HKWF1971/2007 H7N7
A/goose/Nebraska/17097-4/2011 H7N9 *
A/guinea fowl/Nebraska/17096-1/2011 H7N9 *
A/n shoveler/Cal/44287 164/2007 H7N7
A/chicken/Arkansas/10/2008 H7N3
A/env/Cal/7451/2010 H7N3
A/env/Maryland/1626/2006 H7N3
A/env/Maryland/276/2006 H7N3
A/env/Maryland/1637/2006 H7N3
A/env/Maryland/261/2006 H7N3
A/env/Maryland/1630/2006 H7N3
A/env/Maryland/1631/2006 H7N3
A/env/Maryland/267/2006 H7N3
A/mallard/Alaska/44430 125/2008 H7N3
A/mallard/Cal/1390/2010 H7N5
A/g wg teal/Cal/11275/2008 H7N3
A/g wg teal/Cal/1841/2009 H7N3
A/rud turnstone/Del Bay/123/2007 H7N3
A/rud turnstone/Del Bay/124/2007 H7N3
A/rud turnstone/Del Bay/121/2007 H7N3
A/rud turnstone/Del Bay/108/2007 H7N3
A/mallard/Alaska/44430 052/2008 H7N2
A/mallard/Del/418/2005 H7N3
A/Ame black dk/NB/2538/2007 H7N3
A/n shoveler/NC/6412 052/2005 H7N6
A/n shoveler/N Caro/6412 050/2005 H7N3
A/n shoveler/N Caro/674 516/2005 H7N3
A/rud turnstone/Del/760/2006 H7N3
A/rud turnstone/NJ/612/2006 H7N3
A/rud turnstone/NJ/563/2006 H7N2
A/rud turnstone/Del Bay/279/2006 H7N3
A/rud turnstone/Del Bay/281/2006 H7N3
A/rud turnstone/Del Bay/282/2006 H7N3
A/rud turnstone/Del Bay/283/2006 H7N3
A/rud turnstone/Del/778/2006 H7N3
A/rud turnstone/NJ/179/2006 H7N3
A/rud turnstone/NJ/196/2006 H7N3
A/shorebird/Del Bay/552/2006 H7N3
0.005

B

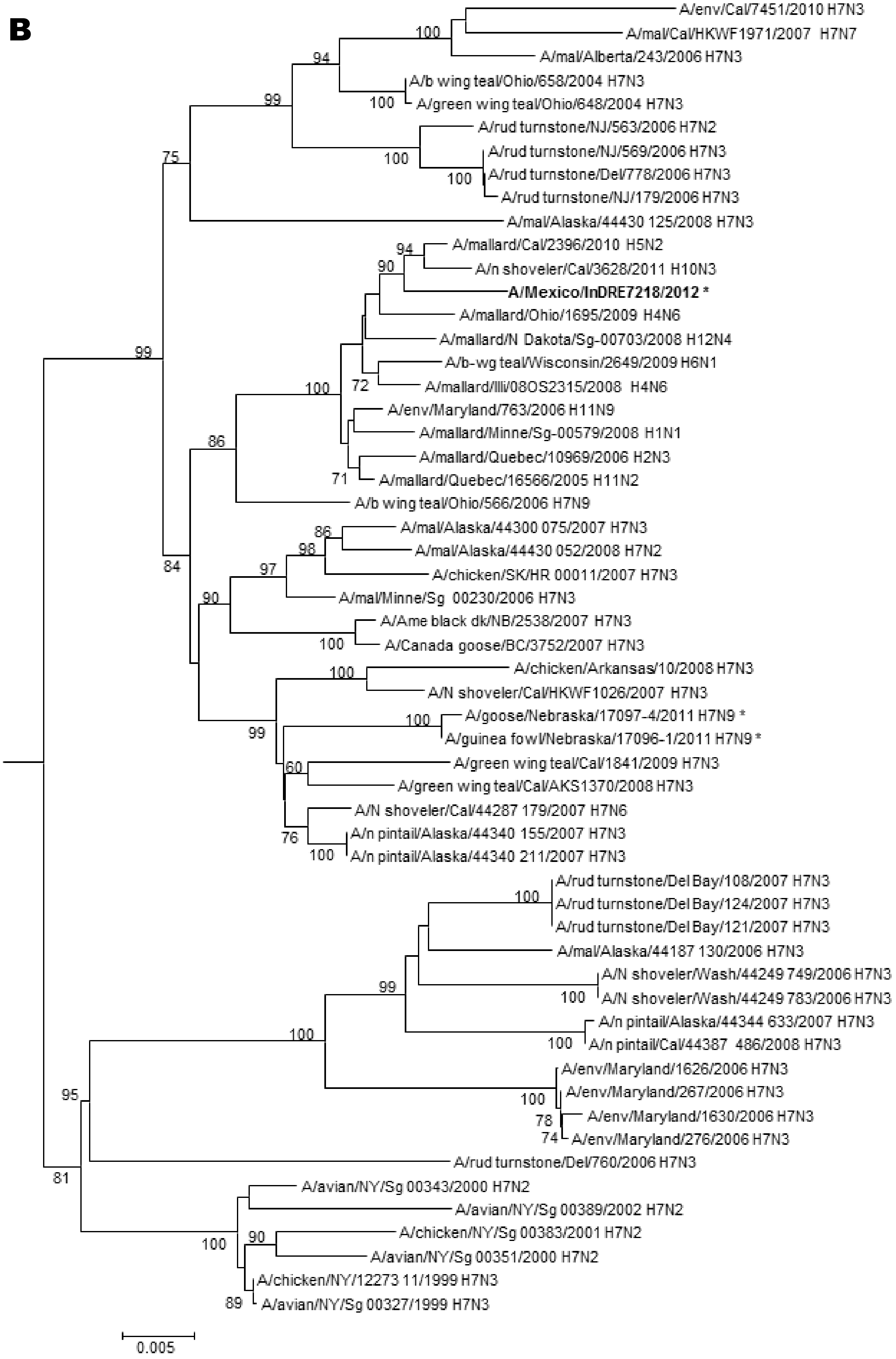
A/env/Cal/7451/2010 H7N3
A/mal/Cal/HKWF1971/2007 H7N7
A/mal/Alberta/243/2006 H7N3
A/b wing teal/Ohio/658/2004 H7N3
A/green wing teal/Ohio/648/2004 H7N3
A/rud turnstone/NJ/563/2006 H7N2
A/rud turnstone/NJ/569/2006 H7N3
A/rud turnstone/Del/778/2006 H7N3
A/rud turnstone/NJ/179/2006 H7N3
A/mal/Alaska/44430 125/2008 H7N3
A/mallard/Cal/2396/2010 H5N2
A/n shoveler/Cal/3628/2011 H10N3
A/Mexico/InDRE7218/2012 *
A/mallard/Ohio/1695/2009 H4N6
A/mallard/N Dakota/Sg-00703/2008 H12N4
A/b-wg teal/Wisconsin/2649/2009 H6N1
A/mallard/Illi/08OS2315/2008 H4N6
A/env/Maryland/763/2006 H11N9
A/mallard/Minne/Sg-00579/2008 H1N1
A/mallard/Quebec/10969/2006 H2N3
A/mallard/Quebec/16566/2005 H11N2
A/b wing teal/Ohio/566/2006 H7N9
A/mal/Alaska/44300 075/2007 H7N3
A/mal/Alaska/44430 052/2008 H7N2
A/chicken/SK/HR 00011/2007 H7N3
A/mal/Minne/Sg 00230/2006 H7N3
A/Ame black dk/NB/2538/2007 H7N3
A/Canada goose/BC/3752/2007 H7N3
A/chicken/Arkansas/10/2008 H7N3
A/N shoveler/Cal/HKWF1026/2007 H7N3
A/goose/Nebraska/17097-4/2011 H7N9 *
A/guinea fowl/Nebraska/17096-1/2011 H7N9 *
A/green wing teal/Cal/1841/2009 H7N3
A/green wing teal/Cal/AKS1370/2008 H7N3
A/N shoveler/Cal/44287 179/2007 H7N6
A/n pintail/Alaska/44340 155/2007 H7N3
A/n pintail/Alaska/44340 211/2007 H7N3
A/rud turnstone/Del Bay/108/2007 H7N3
A/rud turnstone/Del Bay/124/2007 H7N3
A/rud turnstone/Del Bay/121/2007 H7N3
A/mal/Alaska/44187 130/2006 H7N3
A/N shoveler/Wash/44249 749/2006 H7N3
A/N shoveler/Wash/44249 783/2006 H7N3
A/n pintail/Alaska/44344 633/2007 H7N3
A/n pintail/Cal/44387 486/2008 H7N3
A/env/Maryland/1626/2006 H7N3
A/env/Maryland/267/2006 H7N3
A/env/Maryland/1630/2006 H7N3
A/env/Maryland/276/2006 H7N3
A/rud turnstone/Del/760/2006 H7N3
A/avian/NY/Sg 00343/2000 H7N2
A/avian/NY/Sg 00389/2002 H7N2
A/chicken/NY/Sg 00383/2001 H7N2
A/avian/NY/Sg 00351/2000 H7N2
A/chicken/NY/12273 11/1999 H7N3
A/avian/NY/Sg 00327/1999 H7N3
0.005

c

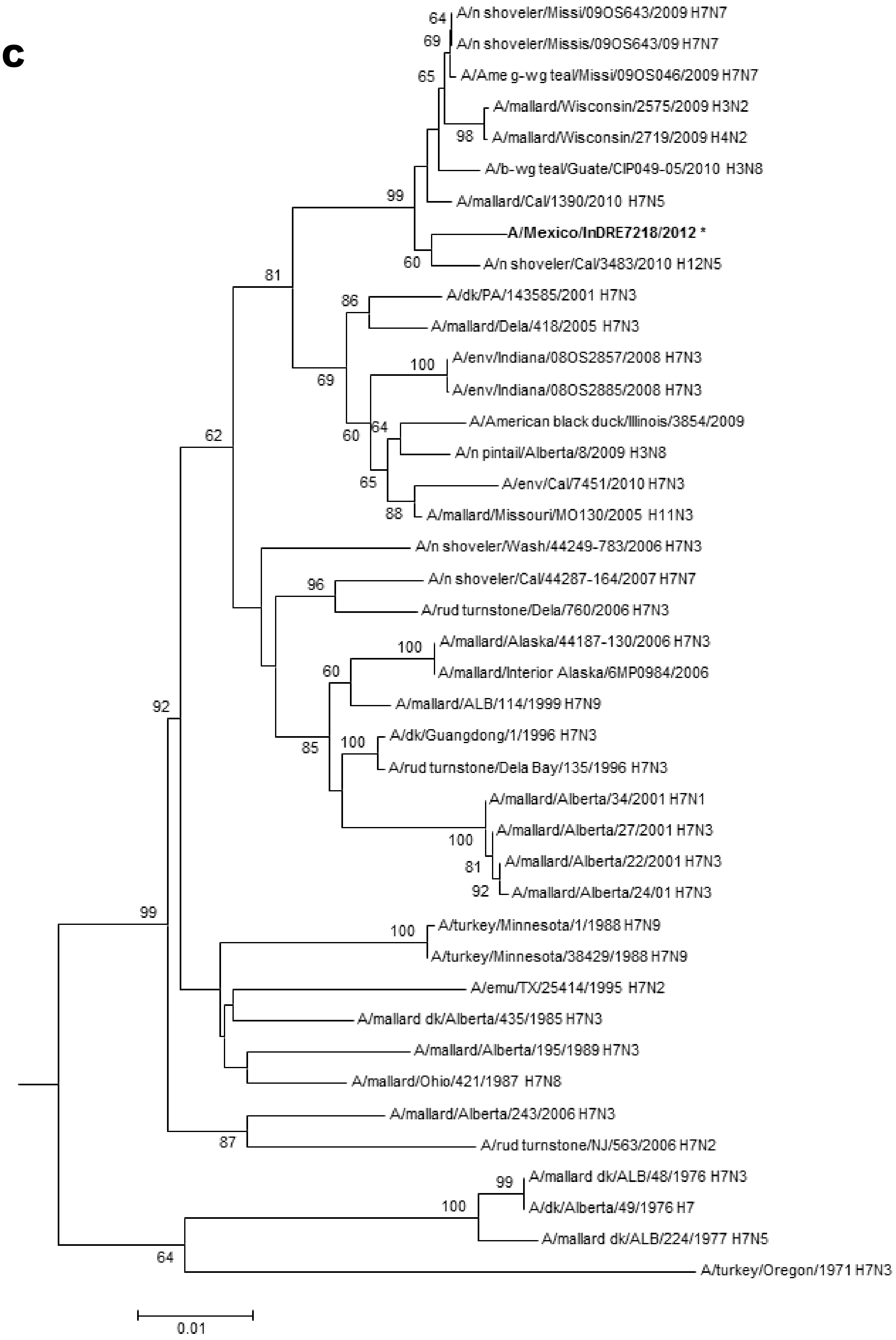
A/n shoveler/Missi/09OS643/2009 H7N7
A/n shoveler/Missis/09OS643/09 H7N7
A/Ame g-wg teal/Missi/09OS046/2009 H7N7
A/mallard/Wisconsin/2575/2009 H3N2
A/mallard/Wisconsin/2719/2009 H4N2
A/b-wg teal/Guate/CIP049-05/2010 H3N8
A/mallard/Cal/1390/2010 H7N5
A/Mexico/InDRE7218/2012 *
A/n shoveler/Cal/3483/2010 H12N5
A/dk/PA/143585/2001 H7N3
A/mallard/Dela/418/2005 H7N3
A/env/Indiana/08OS2857/2008 H7N3
A/env/Indiana/08OS2885/2008 H7N3
A/American black duck/Illinois/3854/2009
A/n pintail/Alberta/8/2009 H3N8
A/env/Cal/7451/2010 H7N3
A/mallard/Missouri/MO130/2005 H11N3
A/n shoveler/Wash/44249-783/2006 H7N3
A/n shoveler/Cal/44287-164/2007 H7N7
A/rud turnstone/Dela/760/2006 H7N3
A/mallard/Alaska/44187-130/2006 H7N3
A/mallard/Interior Alaska/6MP0984/2006
A/mallard/ALB/114/1999 H7N9
A/dk/Guangdong/1/1996 H7N3
A/rud turnstone/Dela Bay/135/1996 H7N3
A/mallard/Alberta/34/2001 H7N1
A/mallard/Alberta/27/2001 H7N3
A/mallard/Alberta/22/2001 H7N3
A/mallard/Alberta/24/01 H7N3
A/turkey/Minnesota/1/1988 H7N9
A/turkey/Minnesota/38429/1988 H7N9
A/emu/TX/25414/1995 H7N2
A/mallard dk/Alberta/435/1985 H7N3
A/mallard/Alberta/195/1989 H7N3
A/mallard/Ohio/421/1987 H7N8
A/mallard/Alberta/243/2006 H7N3
A/rud turnstone/NJ/563/2006 H7N2
A/mallard dk/ALB/48/1976 H7N3
A/dk/Alberta/49/1976 H7
A/mallard dk/ALB/224/1977 H7N5
A/turkey/Oregon/1971 H7N3
0.01

D

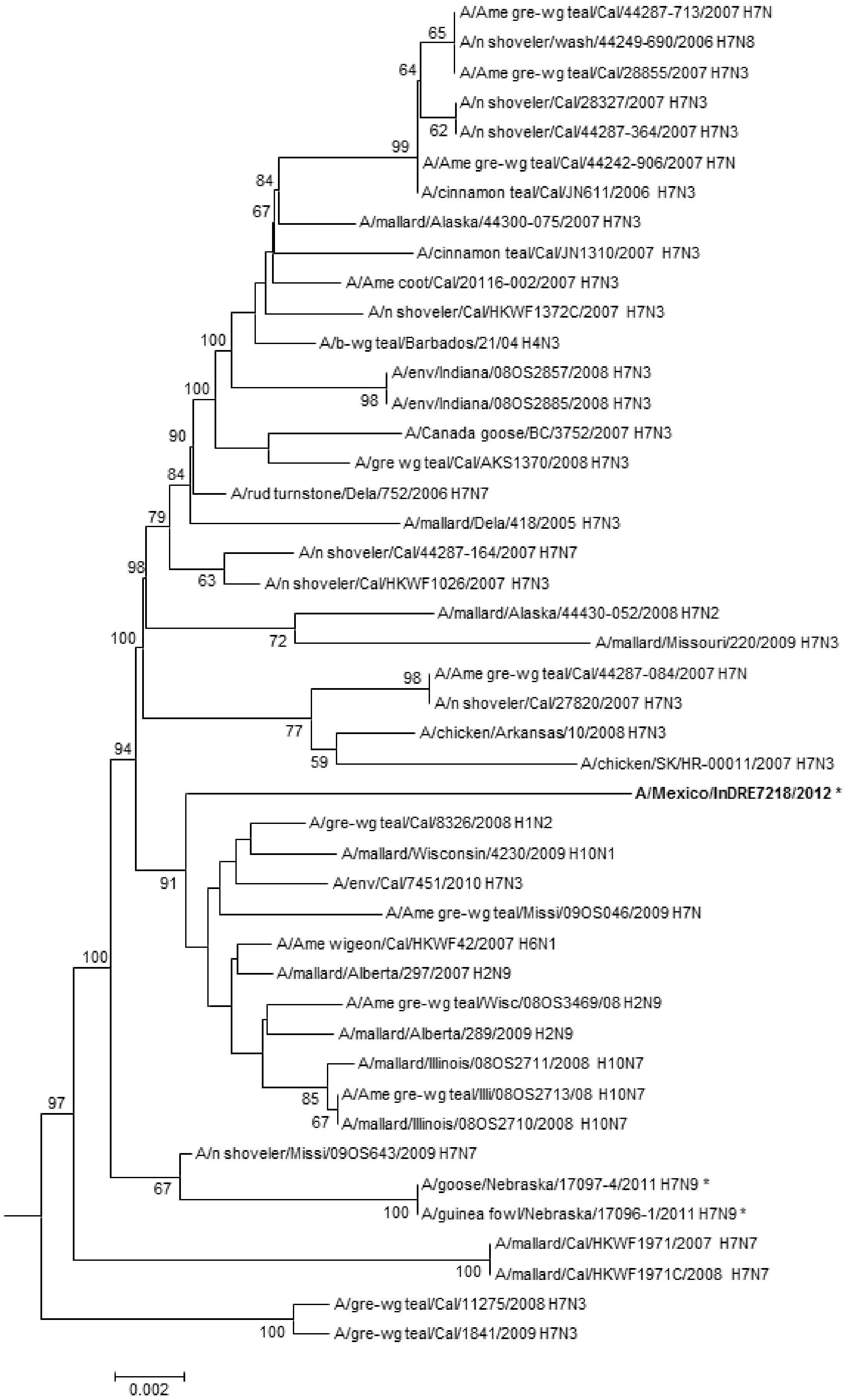
A/Ame gre-wg teal/Cal/44287-713/2007 H7N
A/n shoveler/wash/44249-690/2006 H7N8
A/Ame gre-wg teal/Cal/28855/2007 H7N3
A/n shoveler/Cal/28327/2007 H7N3
A/n shoveler/Cal/44287-364/2007 H7N3
A/Ame gre-wg teal/Cal/44242-906/2007 H7N
A/cinnamon teal/Cal/JN611/2006 H7N3
A/mallard/Alaska/44300-075/2007 H7N3
A/cinnamon teal/Cal/JN1310/2007 H7N3
A/Ame coot/Cal/20116-002/2007 H7N3
A/n shoveler/Cal/HKWF1372C/2007 H7N3
A/b-wg teal/Barbados/21/04 H4N3
A/env/Indiana/08OS2857/2008 H7N3
A/env/Indiana/08OS2885/2008 H7N3
A/Canada goose/BC/3752/2007 H7N3
A/gre wg teal/Cal/AKS1370/2008 H7N3
A/rud turnstone/Dela/752/2006 H7N7
A/mallard/Dela/418/2005 H7N3
A/n shoveler/Cal/44287-164/2007 H7N7
A/n shoveler/Cal/HKWF1026/2007 H7N3
A/mallard/Alaska/44430-052/2008 H7N2
A/mallard/Missouri/220/2009 H7N3
A/Ame gre-wg teal/Cal/44287-084/2007 H7N
A/n shoveler/Cal/27820/2007 H7N3
A/chicken/Arkansas/10/2008 H7N3
A/chicken/SK/HR-00011/2007 H7N3
A/Mexico/InDRE7218/2012 *
A/gre-wg teal/Cal/8326/2008 H1N2
A/mallard/Wisconsin/4230/2009 H10N1
A/env/Cal/7451/2010 H7N3
A/Ame gre-wg teal/Missi/09OS046/2009 H7N
A/Ame wigeon/Cal/HKWF42/2007 H6N1
A/mallard/Alberta/297/2007 H2N9
A/Ame gre-wg teal/Wisc/08OS3469/08 H2N9
A/mallard/Alberta/289/2009 H2N9
A/mallard/Illinois/08OS2711/2008 H10N7
A/Ame gre-wg teal/Illi/08OS2713/08 H10N7
A/mallard/Illinois/08OS2710/2008 H10N7
A/n shoveler/Missi/09OS643/2009 H7N7
A/goose/Nebraska/17097-4/2011 H7N9 *
A/guinea fowl/Nebraska/17096-1/2011 H7N9 *
A/mallard/Cal/HKWF1971/2007 H7N7
A/mallard/Cal/HKWF1971C/2008 H7N7
A/gre-wg teal/Cal/11275/2008 H7N3
A/gre-wg teal/Cal/1841/2009 H7N3
0.002

E

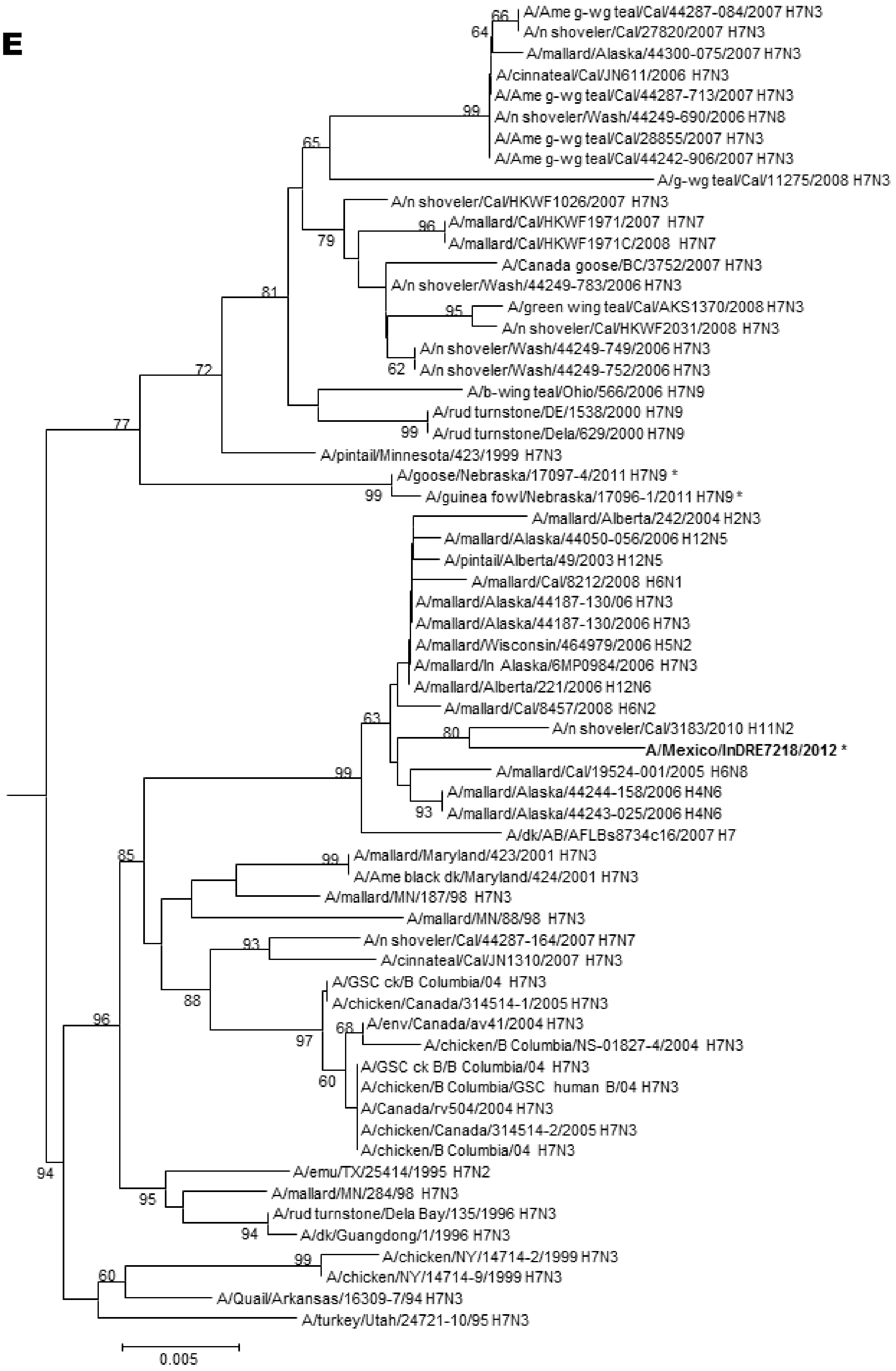
A/Ame g-wg teal/Cal/44287-084/2007 H7N3
A/n shoveler/Cal/27820/2007 H7N3
A/mallard/Alaska/44300-075/2007 H7N3
A/cinnateal/Cal/JN611/2006 H7N3
A/Ame g-wg teal/Cal/44287-713/2007 H7N3
A/n shoveler/Wash/44249-690/2006 H7N8
A/Ame g-wg teal/Cal/28855/2007 H7N3
A/Ame g-wg teal/Cal/44242-906/2007 H7N3
A/g-wg teal/Cal/11275/2008 H7N3
A/n shoveler/Cal/HKWF1026/2007 H7N3
A/mallard/Cal/HKWF1971/2007 H7N7
A/mallard/Cal/HKWF1971C/2008 H7N7
A/Canada goose/BC/3752/2007 H7N3
A/n shoveler/Wash/44249-783/2006 H7N3
A/green wing teal/Cal/AKS1370/2008 H7N3
A/n shoveler/Cal/HKWF2031/2008 H7N3
A/n shoveler/Wash/44249-749/2006 H7N3
A/n shoveler/Wash/44249-752/2006 H7N3
A/b-wing teal/Ohio/566/2006 H7N9
A/rud turnstone/DE/1538/2000 H7N9
A/rud turnstone/Dela/629/2000 H7N9
A/pintail/Minnesota/423/1999 H7N3
A/goose/Nebraska/17097-4/2011 H7N9 *
A/guinea fowl/Nebraska/17096-1/2011 H7N9 *
A/mallard/Alberta/242/2004 H2N3
A/mallard/Alaska/44050-056/2006 H12N5
A/pintail/Alberta/49/2003 H12N5
A/mallard/Cal/8212/2008 H6N1
A/mallard/Alaska/44187-130/06 H7N3
A/mallard/Alaska/44187-130/2006 H7N3
A/mallard/Wisconsin/464979/2006 H5N2
A/mallard/ln Alaska/6MP0984/2006 H7N3
A/mallard/Alberta/221/2006 H12N6
A/mallard/Cal/8457/2008 H6N2
A/n shoveler/Cal/3183/2010 H11N2
A/Mexico/InDRE7218/2012 *
A/mallard/Cal/19524-001/2005 H6N8
A/mallard/Alaska/44244-158/2006 H4N6
A/mallard/Alaska/44243-025/2006 H4N6
A/dk/AB/AFLBs8734c16/2007 H7
A/mallard/Maryland/423/2001 H7N3
A/Ame black dk/Maryland/424/2001 H7N3
A/mallard/MN/187/98 H7N3
A/mallard/MN/88/98 H7N3
A/n shoveler/Cal/44287-164/2007 H7N7
A/cinnateal/Cal/JN1310/2007 H7N3
A/GSC ck/B Columbia/04 H7N3
A/chicken/Canada/314514-1/2005 H7N3
A/env/Canada/av41/2004 H7N3
A/chicken/B Columbia/NS-01827-4/2004 H7N3
A/GSC ck B/B Columbia/04 H7N3
A/chicken/B Columbia/GSC human B/04 H7N3
A/Canada/rv504/2004 H7N3
A/chicken/Canada/314514-2/2005 H7N3
A/chicken/B Columbia/04 H7N3
A/emu/TX/25414/1995 H7N2
A/mallard/MN/284/98 H7N3
A/rud turnstone/Dela Bay/135/1996 H7N3
A/dk/Guangdong/1/1996 H7N3
A/chicken/NY/14714-2/1999 H7N3
A/chicken/NY/14714-9/1999 H7N3
A/Quail/Arkansas/16309-7/94 H7N3
A/turkey/Utah/24721-10/95 H7N3
0.005

F

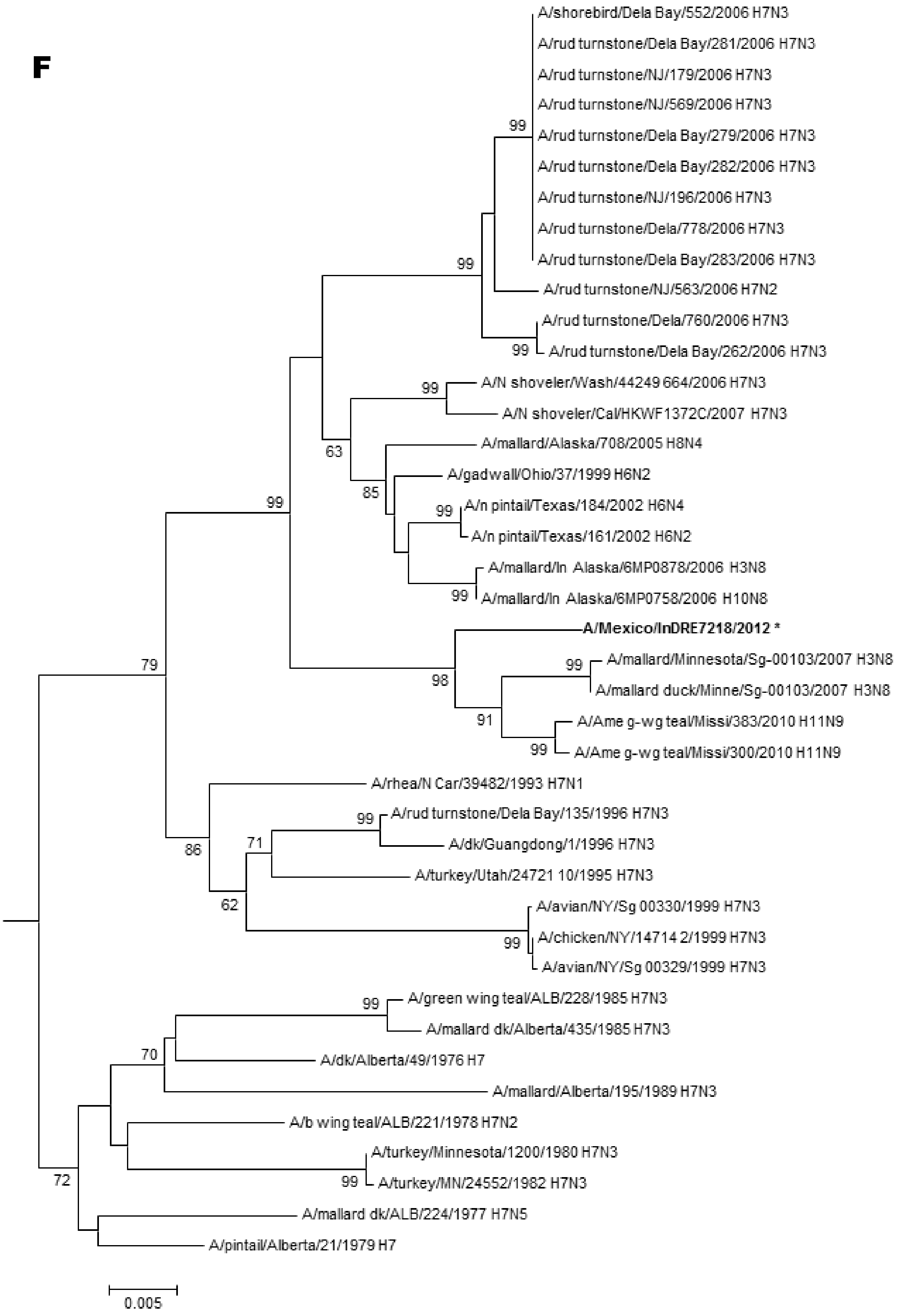
A/shorebird/Dela Bay/552/2006 H7N3
A/rud turnstone/Dela Bay/281/2006 H7N3
A/rud turnstone/NJ/179/2006 H7N3
A/rud turnstone/NJ/569/2006 H7N3
A/rud turnstone/Dela Bay/279/2006 H7N3
A/rud turnstone/Dela Bay/282/2006 H7N3
A/rud turnstone/NJ/196/2006 H7N3
A/rud turnstone/Dela/778/2006 H7N3
A/rud turnstone/Dela Bay/283/2006 H7N3
A/rud turnstone/NJ/563/2006 H7N2
A/rud turnstone/Dela/760/2006 H7N3
A/rud turnstone/Dela Bay/262/2006 H7N3
A/N shoveler/Wash/44249 664/2006 H7N3
A/N shoveler/Cal/HKWF1372C/2007 H7N3
A/mallard/Alaska/708/2005 H8N4
A/gadwall/Ohio/37/1999 H6N2
A/n pintail/Texas/184/2002 H6N4
A/n pintail/Texas/161/2002 H6N2
A/mallard/In Alaska/6MP0878/2006 H3N8
A/mallard/In Alaska/6MP0758/2006 H10N8
A/Mexico/InDRE7218/2012 *
A/mallard/Minnesota/Sg-00103/2007 H3N8
A/mallard duck/Minne/Sg-00103/2007 H3N8
A/Ame g-wg teal/Missi/383/2010 H11N9
A/Ame g-wg teal/Missi/300/2010 H11N9
A/rhea/N Car/39482/1993 H7N1
A/rud turnstone/Dela Bay/135/1996 H7N3
A/dk/Guangdong/1/1996 H7N3
A/turkey/Utah/24721 10/1995 H7N3
A/avian/NY/Sg 00330/1999 H7N3
A/chicken/NY/14714 2/1999 H7N3
A/avian/NY/Sg 00329/1999 H7N3
A/green wing teal/ALB/228/1985 H7N3
A/mallard dk/Alberta/435/1985 H7N3
A/dk/Alberta/49/1976 H7
A/mallard/Alberta/195/1989 H7N3
A/b wing teal/ALB/221/1978 H7N2
A/turkey/Minnesota/1200/1980 H7N3
A/turkey/MN/24552/1982 H7N3
A/mallard dk/ALB/224/1977 H7N5
A/pintail/Alberta/21/1979 H7
99
99
99
99
63
85
99
99
99
98
91
99
79
86
71
99
62
99
99
70
99
72
0.005
